# Comparing Conscious and Unconscious Conflict Adaptation

**DOI:** 10.1371/journal.pone.0055976

**Published:** 2013-02-06

**Authors:** Kobe Desender, Elke Van Lierde, Eva Van den Bussche

**Affiliations:** Department of Psychology, Vrije Universiteit Brussel, Brussels, Belgium; Radboud University Nijmegen, The Netherlands

## Abstract

Recently, researchers have been trying to unravel the function of consciousness by exploring whether unconscious information is (in)capable of exerting cognitive control. Theoretically, cognitive control functions, such as conflict adaptation, have often been assumed to require consciousness. However, empirical evidence on conscious versus unconscious conflict adaptation is highly contradictory and hitherto, only one study reliably demonstrated adaptation to unconscious conflict. Therefore, the current study wanted to shed further light on this debated issue. A masked and unmasked version of the priming paradigm were used to create unconscious and conscious conflict trials (i.e., when prime and target trigger opposite responses). In contrast to previous studies, the Stimulus Onset Asynchrony was kept constant in both conditions and neutral trials were added to the design in order to investigate the origin of the adaptation and to investigate the specific adaptation effects. Our results showed robust conflict adaptation effects following conscious and unconscious conflict. Furthermore, our results suggest that the adaptation elicited by the conflict, is mainly an adaptation of interference, not of facilitation. We can conclude that conflict adaptation can occur after unconscious conflict, which indicates that this expression of cognitive control is most likely not an exclusive function of consciousness.

## Introduction

### The function of consciousness

Generations of scientists and philosophers have struggled with the mystery of the functions of consciousness in cognitive processing. Despite a large number of studies addressing this issue, no consensus has been reached. It is becoming increasingly clear that unconscious information processing can reach a sophisticated cognitive level and shares many features and processing routes with conscious processing [1]–[3]. For example, even shortly presented and heavily masked stimuli, which are never consciously perceived, can be processed up to a high semantic level [1], [4] and unconscious processing is susceptible to several conscious top-down modulations [5], [6]. Based on these findings, researchers began to wonder whether consciousness has a special function at all [7], [8]. Several theoretical frameworks postulate that *cognitive control*, which allows us to be flexible and adjust our behavior to different contexts and goals [9]–[11], is exclusively associated with consciousness [12]–[14]. For example, according to the Global Neuronal Workspace theory [15], [16], unconscious stimuli can be processed by specialized modular systems. However, without global ignition (the determinant of consciousness), an unconscious stimulus should be unable to initiate top-down cognitive control, since it remains within a modular system. Consequently, cognitive control operations such as planning a new strategy, evaluating it, controlling its execution and correcting possible errors would require conscious access. Contrarily, others argue for the possibility of unconscious cognitive control. For example, based on the unconscious goal pursuit literature [17], higher cognitive processes underlying goal pursuit are proposed not to require consciousness [18], [19]. It becomes clear that exploring whether unconscious information is (in)capable of exerting cognitive control provides a fruitful approach to explore the function of consciousness.

### Conflict adaptation

Cognitive control kicks in when routine activation of behavior is no longer sufficient for optimal performance [11]. When people encounter interference they adjust their behavior to overcome it. This interference can take various forms. For example, in a situation where relevant and irrelevant information can activate differential responses, this potential response conflict requires remedial action [20]. In the current study we will focus on this particular expression of cognitive control, known as *conflict adaptation*. To study this effect we used a priming paradigm in which subjects are instructed to categorize a target (i.e., the relevant information) as fast as possible, while ignoring a preceding prime (i.e., the irrelevant information). When prime and target trigger the same response (i.e., congruent trial) responses are typically fast and error rates low. However, when prime and target trigger a different response (i.e., incongruent trial), both sources are highly conflicting, which typically leads to slower response times and elevated error rates. The interesting observation is that subjects continuously adapt to this conflicting information. When they experience a conflict on the previous trial, they will react to this by reducing the detrimental influence of the irrelevant information, leading to reduced *priming effects* (i.e., faster responses to congruent compared to incongruent trials) on the current trial [21]. This is achieved by inhibiting irrelevant information and/or focusing on relevant information [22]. This effect, also known as the Gratton effect, is typically calculated by computing the difference between congruency effects following congruent and following incongruent trials [23]. It is a highly robust finding, independent of the particular paradigm being used [24].

### Unconscious conflict adaptation?

In recent years, several authors have suggested that consciousness of the conflicting information is a prerequisite in order to be able to adapt to the conflict (for an extensive discussion of this topic, see [25]). For example, Kunde [21] showed conflict adaptation with perfectly visible primes. However, when using metacontrast masking, so that subjects could not consciously perceive the primes, this effect vanished completely. According to these results, awareness of the conflicting information is necessary for conflict adaptation (for similar findings, see [26]–[28]). In contrast to these studies, van Gaal and colleagues [29] recently observed unconscious conflict adaptation. They reasoned that Kunde [21] failed to observe unconscious adaptation because he used rather long inter-trial intervals, which might have destroyed weak unconscious traces [30]. In their study, two elements in the design of Kunde [21] were changed; the inter-trial interval was shortened and an auditory warning signal prior to each trial was eliminated. With these slight variations, van Gaal et al. [29] observed reliable conflict adaptation following both conscious and unconscious incongruent trials. To date, this is the only study providing convincing evidence for the possibility of truly unconscious conflict adaptation. Although two other recent studies hinted at the possibility of unconscious conflict adaptation [31], [32], they were both subject to critique [25].

Given these highly contradictory results, we will aim to provide further clarification in the current study. First, because only one single study observed reliable unconscious conflict adaptation, there is an apparent need to replicate this finding. Second, because van Gaal et al. [29] manipulated prime visibility by using different Stimulus-Onset Asynchronies (SOA) in the unconscious and conscious condition, their observation that conflict adaptation was significantly smaller in the unconscious condition compared to the conscious condition might have been caused by this difference in SOA. They found that short SOA (i.e., unconscious) trials yielded small congruency effects, and consequently the conflict-evoking capacity of those trials was weaker than that of trials with a longer (i.e., conscious) SOA. Furthermore, since their short and long SOA trials were presented intermixed instead of blocked, this could also have decreased the observed unconscious conflict adaptation [33]. An additional disadvantage of presenting conscious and unconscious trials intermixed is that subjects are fully aware that primes are presented throughout the experiment, which could have raised sensitivity for unconscious primes. Ideally, we would want to know if subjects can unconsciously adapt to conflicting primes, even when they are completely unaware that primes are presented at all. Moreover, if subjects are completely unaware of the presence of the unconscious primes, they cannot, as would be the case in a mixed design, strategically handle the unconscious trials based on their knowledge of the conscious trials. Therefore, in the present study, we will keep the SOA constant in the unconscious and conscious condition and always first present a block with masked primes, and second a block with clearly visible primes. Interestingly, a recent study matched for these differences in SOA, and presented conscious and unconscious primes in separate blocks, and nevertheless failed to observe unconscious conflict adaptation [26]. However, these authors used a trial-by-trial prime visibility assessment which might have preserved strong neural traces of conscious primes, but wiped out the weak, short-lived traces of unconscious primes [30]. This probably resulted in the absence of unconscious conflict adaptation.

Third, we added neutral trials to the design, in order to examine *the origin* of conflict adaptation. When only congruent and incongruent trials are used, it cannot be ruled out that the decrease of the congruency effect following an incongruent trial is in fact an increase of the effect following a congruent trial [34]. Using neutral trials as a baseline, we can disentangle these different alternatives and the role of consciousness in these processes. Because neutral trials do not facilitate or interfere with responses to the target, we expect no adaptation following these trials. If adaptation is selectively driven by conflict, we thus expect congruency effects to be reduced following incongruent trials compared to neutral and congruent trials. Importantly, we expect no difference between the latter two. On the other hand, if adaptation is selectively driven by congruent trials, we expect congruency effects to be enhanced following congruent trials, compared to neutral or incongruent trials. The latter two should not differ here. Finally, if we would find that congruency effects are reduced following incongruent trials, and enhanced following congruent trials, both compared to neutral trials, this would suggest that both trial types add to the effect.

Apart from the origin of adaptation, another interesting question is which specific *adaptation processes* are evoked. In a design with neutral trials, it can be examined which particular type of trial (congruent or incongruent) is most affected by the adaptation elicited on the previous trial. Is the sequential modulation of the congruency effect the result of adaptation of facilitation (i.e., faster responses on congruent trials than on neutral trials) following conflict trials, or is it the result of adaptation of interference (i.e., slower responses on incongruent trials than on neutral trials) following conflict trials. Furthermore, we can examine whether these specific adaptation processes (i.e., adaptation of facilitation and adaptation of interference) differ between the conscious and unconscious condition.

## Methods

### Ethics statement

All procedures were executed in compliance with relevant laws and institutional guidelines. Subjects participated as partial fulfillment of a course requirement. Participants received experimental regulations which stipulated [translated from Dutch]: “Each student is required to read the “Informed Consent” prior to participation. In this Consent, the content of the experiment and the inclusion criteria for the participants are mentioned. In this “Informed Consent” it is always mentioned that you, as a participant, are aware of the content of these regulations and that you agree with them”. Since the data were analyzed completely anonymously (i.e., from the start of the experiment we refrained from registering the participants' names), participants gave oral informed consent before experimentation and signed an attendance list afterwards. They were invited to a debriefing session. The Medical Ethics Committee of the Vrije Universiteit Brussel was consulted and based on our full protocol (including the consent procedure) they decided that our study was exempt from approval (reference 2012/205).

### Participants

Sixty-five students of the Vrije Universiteit Brussel (VUB) participated in this study. Two participants were eliminated because they made more than 20% errors in the masked condition. Two participants were eliminated because reaction times were more than two standard deviations (*SD*) above the average mean on the unconscious condition; three participants were eliminated because reaction times were more than two standard deviations (*SD*) above the average mean on the conscious condition; two participants were eliminated because reaction times were more than two standard deviations (*SD*) above the average mean on both conditions. Thus, the final sample consisted of 56 participants (49 females), with an age range of 17 to 26 years (*M* = 19.0, *SD* = 1.7). All participants had normal or corrected-to-normal vision. They participated in exchange for course credit.

### Apparatus and stimuli

The experiment was run on Intel Pentium 4 computers with 17 inch LCD screens. The refresh rate was set to 60 Hz and stimulus presentation was synchronized with the vertical refresh rate (16.7 ms). E-prime version 1.1. was used for stimulus presentation and data collection. The data were analyzed using SPSS 19.

Targets were the Arabic numbers “1” or “9”. Primes were the Arabic numbers “1” or “9” or the neutral prime “X”. The forward mask was “#$#” and the backward mask was “$#$”. All stimuli were presented in white on a black background in the center of the screen. The used font was Arial, size 14.

### Procedure

The experiment comprised three parts: a practice block, an experimental block and a posttest to assess the visibility of the primes. All participants had to complete these three parts twice, once in the unconscious and once in the conscious condition. To prevent that subjects became aware of the presence of the primes in the unconscious condition, this condition was always carried out first.

**Figure 1:**
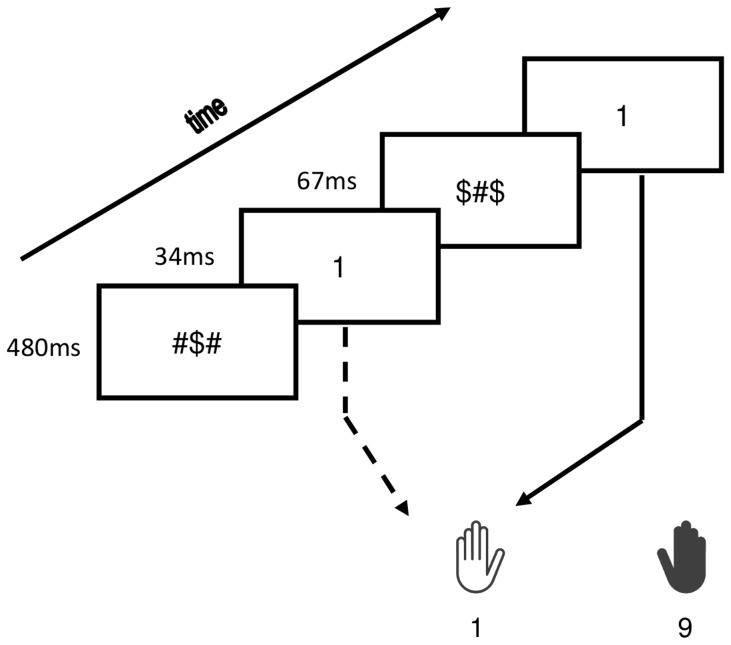
Experimental procedure. Example of a congruent trial in the masked condition.

Each trial started with a forward mask presented for 480 ms. Then a prime appeared for 33 ms. Afterwards, a backward mask (in the unconscious condition) or a blank screen (in the conscious condition) appeared for 67 ms. Finally the target was presented until a response was made. Participants were instructed to categorize the target as quickly and accurately as possible. They had to respond on a qwerty-keyboard, by pressing “q” with the left index finger in response to “1” and “p” with the right index finger in response to “9”. The inter-stimulus interval was set to 1000 ms. Figure 1 shows an example of an experimental trial.

Participants began with the unconscious condition. They first received eight practice trials, during which no prime was shown. Afterwards, they were presented with 180 randomly selected experimental trials with an equal amount of congruent, incongruent and neutral trials. After the experimental trials, participants were informed about the presence of the primes and were asked to participate in a posttest where they had to categorize the prime as “1” or “9”, instead of the target. They received 40 trials, similar to the experimental trials with the exclusion of neutral trials. They were instructed to perform the task at their own pace. Whenever they were not sure about the identity of the prime, they were forced to guess. After completing the practice block, experimental block and posttest of the unconscious condition, participants received the same three parts of the conscious condition.

## Results

### Reaction times

Reaction times (RTs) above 1000 ms (0.005% of the data) and trials following an error (4.2%) were excluded from further analysis. Mean reaction times of correct trials were subjected to a repeated measures ANOVA with congruency on the current trial (3 levels: congruent, neutral or incongruent), congruency on the previous trial (3 levels: congruent, neutral or incongruent) and condition (2 levels: conscious or unconscious) as within-subject factors. Mean RTs as a function of these factors are listed in Table 1.

**Table 1:** Mean reaction times (SD) and error rates (SD) in both conditions as a function of congruency on the previous and the current trial.

		Current trial			Priming	Facilitation	Interference
Condition	Previous trial	Congruent	Neutral	Incongruent			
Unconscious	Congruent	424 (33.4)	428 (32.6)	444 (30.4)	20***	4	16***
		1.05 (2.86)	3,15 (5,11)	3,74 (5,17)	2.7**	2.1**	0.6
	Neutral	427 (29.4)	426 (38.8)	443 (30.5)	16***	−1	17***
		2,69 (4,02)	1,87 (4,31)	4,65 (6,02)	2.0*	−0.8	2.8**
	Incongruent	429 (32.6)	429 (32.1)	437 (34.3)	8**	0	8**
		2,84 (3,86)	1,92 (3,63)	2,92 (4,79)	0.1	−0.9	1
Conscious	Congruent	396 (48.4)	465 (41.7)	505 (40.8)	109***	69***	40***
		0,84 (2,58)	1,83 (2,86)	13,33 (9,91)	12.5***	1*	11.5***
	Neutral	397 (37.9)	455 (38.3)	499 (44.8)	102***	58***	44***
		0,55 (1,65)	1,01 (3,09)	11,10 (10,82)	10.5***	0.5	10.1***
	Incongruent	411 (45.5)	471 (40.5)	494 (48.7)	83***	60***	23***
		0,79 (2,52)	1,39 (3,09)	7,45 (8,52)	6.7***	0.6	6.1***

*Note*. Priming = Incongruent – Congruent; Facilitation = Neutral – Congruent; Interference = Incongruent – Neutral;

* *p*<.05;

** *p*<.01;

*** *p*<.001.

This analysis showed a main effect of condition (*F*(1,55) = 28.41, *p*<.001), with participants responding on average 23 ms faster in the unconscious compared to the conscious condition. We also observed a main effect of congruency on the current trial (*F*(2,54) = 338.98, *p*<.001): subjects responded significantly faster to congruent trials compared to neutral (414 ms versus 446 ms, *t*(55) = −17.19, *p*<.001) and compared to incongruent (414 ms versus 470 ms, *t*(55) = −26.27, *p*<.001) trials. Subjects also responded significantly faster to neutral trials compared to incongruent trials (446 ms versus 470 ms, *t*(55) = 14.30, *p*<.001). There was also an interaction between condition and congruency on the current trial (*F*(2,54) = 235.25, *p*<.001), reflecting the fact that the differences in RTs depending on whether the current trial was congruent, neutral or incongruent were larger in the conscious than the unconscious condition (average RTs respectively 401 ms, 464 ms and 499 ms in the conscious condition and 427 ms, 428 ms and 441 ms in the unconscious condition). There was also an interaction between condition and congruency on the previous trial (*F*(2,54) = 3.78, *p* = .029), reflecting the fact that the differences in RTs depending on whether the previous trial was congruent, neutral or incongruent were slightly more prominent in the conscious than the unconscious condition (average RTs respectively 455 ms, 450 ms and 459 ms in the conscious condition and 432 ms, 432 ms and 432 ms in the unconscious condition). Crucially, there was an interaction between congruency on the current trial and congruency on the previous trial (*F*(4,52) = 4.96, *p* = .002). Interestingly, this interaction was not modulated by the condition (*F*<1). Separate ANOVAs showed that the interaction was apparent in the conscious condition (*F*(4,52) = 3,71, *p* = .01), but was not statistically significant in the unconscious condition (*F*(4,52) = 1.95, *p* = .12). Note that this does not imply that there was no conflict adaptation in the latter condition. Because of the inclusion of neutral trials this interaction did not reach significance. However, when excluding neutral trials from this analysis, the interaction was, in fact, strongly significant (*F*(1,55) = 7.34, *p* = .009). None of the other effects reached significance.

### Conflict adaptation in reaction times

To interpret the significant two-way interaction between congruency on the current trial and congruency on the previous trial, we examined the influence of the previous trial status on the priming effect (RT_incongruent_ – RT_congruent_), the interference effect (RT_incongruent_ – RT_neutral_), and the facilitation effect (RT_neutral_ – RT_congruent_).

**Figure 2:**
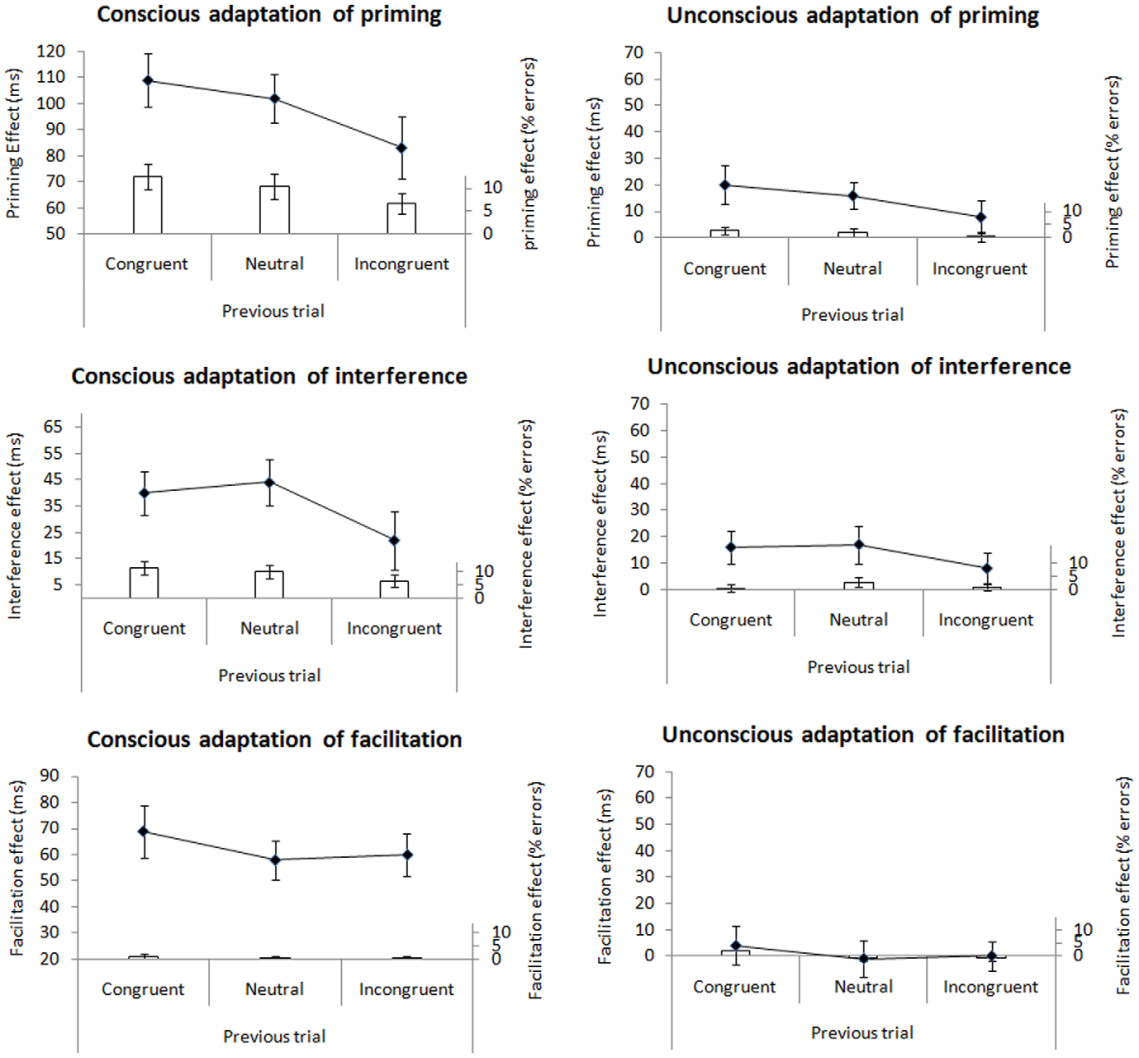
Conflict adaptation results. Priming effects (RT_incongruent_ – RT_congruent_), interference effects (RT_incongruent_ – RT_neutral_), and facilitation effects (RT_neutral_ – RT_congruent_) as a function of congruency on the current and previous trial (congruent, neutral or incongruent) and condition (conscious or unconscious). Lines depict the effects for RTs and bars depict the effects for error rates. Error bars reflect 95% within-subject confidence intervals.

In the conscious condition, the *priming effect* was smaller following an incongruent trial compared to a congruent trial (83 versus 109 ms; *t*(55) = −3.74, *p*<.001). Likewise, in the unconscious condition, the priming effect was smaller following an incongruent trial, compared to a congruent trial (8 versus 20 ms; *t*(55) = −2.71, *p* = .009). The adaptation effect in the conscious condition was marginally larger than the adaptation effect in the unconscious condition (*t*(55) = −1.93, *p* = .058). Thus, in both the conscious and the unconscious condition we observed reliable conflict adaptation, with the conscious conflict adaptation being larger in size (see Figure 2). The priming effect was also significantly smaller following an incongruent trial compared to a neutral trial, both in the conscious (83 versus 102 ms; *t*(55) = −2.73, *p* = .009) and the unconscious condition (8 versus 16 ms; *t*(55) = −2.12, *p* = .039). To examine whether the sequential modulation of the congruency effect can also be explained as an enhancement following congruent trials, we examined whether priming effects were enhanced following congruent trials compared to neutral trials. This, however, was not the case: neither in the conscious (109 versus 102 ms; *p* = .18) nor the unconscious condition (20 versus 16 ms; *p* = .31).

Next to this reduction of the priming effect following an incongruent trial, a reduction of the *interference effect* was apparent in the conscious condition. Here, the interference effect was smaller following an incongruent trial compared to a neutral trial (22 versus 44 ms; *t*(55) = −3.12, *p* = .003). The difference between the interference effect following an incongruent trial compared to a congruent trial also reached significance (22 versus 40 ms; *t*(55) = −2.63, *p* = .011). In the unconscious condition the interference effect was also smaller following an incongruent trial compared to a neutral trial (8 versus 17 ms; *t*(55) = −1.94, *p* = .057) and following an incongruent trial compared to a congruent trial (8 versus 16 ms; *t*(55) = −1.95, *p* = .056), although both reductions were only marginally significant.

When examining the *facilitation effect* dependent on the status of the previous trial, no differences were apparent. In the conscious condition the effect remained rather stable, following an incongruent (60 ms), a congruent (69 ms) or a neutral trial (58 ms; all *p*'s>.07). In the unconscious condition, this effect was also not different following an incongruent (0 ms), a congruent (4 ms) or a neutral trial (−1 ms; all *p*'s>.32). All effects are depicted on Figure 2.

### Error rates

When conducting the same repeated measures analysis on the error rates, the same pattern of results was found. Mean error rates as a function of the factors in the analysis are listed in Table 1.

This analysis showed a main effect of condition (*F*(1,55) = 11.65, *p* = .001), with participants making more errors in the conscious compared to the unconscious condition (4.2% versus 2.7%). We also observed a main effect of congruency on the current trial (*F*(2,54) = 38.43, *p*<.001): subjects made significantly more errors on incongruent trials compared to neutral (7.2% versus 1.9.%, *t*(55) = 8.84, *p*<.001) and congruent (7.2% versus 1.5%, *t*(55) = 8.11, *p*<.001) trials. The amount of errors made on neutral and congruent trials did not differ (1.9% versus 1.5%, *t*(55) = 1.54, *p* = .13). There was also an interaction between condition and congruency on the current trial (*F*(2,54) = 32.63, *p*<.001), reflecting the fact that the increased error rate for incongruent trials compared to neutral and congruent trials was more prominent for the conscious (average error rates respectively 10.6%, 1.41% and 0.7%) than for the unconscious condition (average error rates respectively 3.8%, 2.3% and 2.2%). There was also a main effect of the congruency of the previous trial (*F*(2,55) = 4.87, *p* = .011). Subjects made less errors when the previous trial was incongruent compared to congruent (2.9% versus 3.99%; *t*(55) = 3.07, *p* = .003), or compared to neutral (2.9% versus 3.2%; *t*(55) = 2.26, *p* = .028). There was also an interaction between condition and congruency on the previous trial (*F*(2,54) = 5.65, *p* = .006), reflecting the fact that the differences in error rates depending on whether the previous trial was congruent, neutral or incongruent were slightly more prominent in the conscious than the unconscious condition (average error rates respectively 5.3%, 4.9% and 3.2% in the conscious condition and 2.6%, 3.1% and 2.5% in the unconscious condition). Crucially, there was an interaction between congruency on the current trial and congruency on the previous trial (*F*(4,52) = 6.70, *p*<.001). Contrasting results from the reaction times, this interaction was modulated by the condition (*F*(4,52) = 3.06, *p* = .025). Separate ANOVAs showed that this interaction was apparent in both the conscious condition (*F*(4,52) = 3.36, *p* = .004) and in the unconscious condition (*F*(4,52) = 3.68, *p* = .01). None of the other effects reached significance.

### Conflict adaptation in error rates

To interpret the interaction between congruency on the current trial and congruency on the previous trial, we again examined the influence of the previous trial status on the priming effect (RT_incongruent_ – RT_congruent_), the interference effect (RT_incongruent_ – RT_neutral_), and the facilitation effect (RT_neutral_ – RT_congruent_).

In the conscious condition, the *priming effect* was smaller following an incongruent trial compared to a congruent trial (6.7% versus 12.5%; *t*(55) = 4.07, *p*<.001). Likewise, in the unconscious condition, the priming effect was smaller following an incongruent trial, compared to a congruent trial (0.08% versus 2.7%; *t*(55) = 2.59, *p* = .012). The conflict adaptation effect was only marginally larger in the conscious compared to the unconscious condition (*t*(55)<1.82, *p* = .074). Thus, in both the conscious and the unconscious condition we observed reliable conflict adaptation (see Figure 2). The priming effect was also reduced following an incongruent trial compared to a neutral trial, both in the conscious condition (6.7% versus 10.54%; *t*(55) = 2.96, *p* = .005), and the unconscious condition (0.08% versus 1.96%; *t*(55) = 2.25, *p* = .028). To examine whether the sequential modulation of the congruency effect can also be explained as an enhancement following congruent trials, we examined whether priming effects were enhanced following congruent trials compared to neutral trials. This, however, was not the case: neither in the conscious (12.5% versus 10.54%, *t*(55) = 1.81, *p* = .076) nor the unconscious condition (2.7% versus 1.96%; *t*<1).

Next to this reduction of the priming effect following an incongruent trial, a reduction of the *interference effect* was apparent in the conscious condition. Here, the interference effect was smaller following an incongruent trial compared to a neutral trial (6.1% versus 10.1%; *t*(55) = 3.09,  = .003) or a congruent trial (6.1% versus 11.5%; *t*(55) = 3.96, *p*<.001). In the unconscious condition, the interference effect was not smaller following an incongruent trial compared to following a neutral trial (1% versus 2.79%; *t*(55) = 1.77, *p* = .082) or following a congruent trial (1% versus 0.6%; *t*<1). A paired samples *t*-test comparing this adaptation of interference (i.e., interference effect _post-congruent_ – interference effect _post-incongruent_) in the conscious condition and the absence of it in the unconscious condition proved to be significant (*t*(55) = −3.22, *p* = .002).

When examining the *facilitation effect* dependent on the status of the previous trial, a reduction of this effect was apparent in the unconscious condition. Here, the facilitation effect was smaller (and even reversed) following an incongruent trial compared to a congruent trial (−0.9% versus 2.1%; *t*(55) = 3.30, *p* = .002), but not compared to a neutral trial (−0.9% versus −0.8%; *t*<1). In the conscious condition the effect remained stable, following an incongruent (0.6%), a congruent (1%) or a neutral trial (0.4%; all *t*'s>.1). A paired samples *t*-test comparing this adaptation of facilitation (i.e., facilitation effect _post-congruent_ – facilitation effect _post-incongruent_) in the unconscious condition and the absence of it in the conscious condition proved to be significant (*t*(55) = 2.32, *p* = .024). All effects are depicted on Figure 2.

### Prime visibility

In the *unconscious condition*, the average proportion of correctly categorized primes (53%) was slightly above chance level (*t*(55) = 2.32, *p* = .024). A measure of prime visibility (*d*′) was calculated for each subject. This measure is obtained by treating one level of the response category (i.e., “1”) as signal and the other level (i.e., “9”) as noise. The mean *d*′ value was 0.20, which was significantly different from 0 (*t*(55) = 3.37, *p* = .001). Non-significant correlations were found between the individual *d′* measure and our three measures of interest, namely conflict adaptation (i.e., priming effect _post-congruent_ – priming effect _post-incongruent_; *r* = −0.01, *p* = .99), adaptation of interference (i.e., interference effect _post-congruent_ – interference effect _post-incongruent_; *r* = 0.12, *p* = .39), and adaptation of facilitation (i.e., facilitation effect _post-congruent_ – facilitation effect _post-incongruent_; *r* = −0.12, *p* = .38). Because on a group level, subjects were able to classify the primes above chance level, it might be the case that our results are caused by a subset of subjects who show above chance-performance in the prime visibility test. To rule out this possibility, we ran a split-median on the individual *d*′ values and calculated the analysis again with the subjects with the lowest *d*′ scores. In this group, *d*′ for unconscious primes was sharply reduced and even reversed to −0.15, which was different from chance level performance (*t*(27) = −2.64, *p* = .014). However, the pattern of results for this low *d*′ group remained highly similar. For the RT analyses, we still obtained an interaction between congruency on the current trial and congruency on the previous trial (*F*(4,24) = 6.54, *p* = .001), which was not modulated by the condition (*F*<1). As before, we observed significant unconscious conflict adaptation (20 ms; *t*(27) = 4.12, *p*<.001), and marginally significant unconscious adaptation of facilitation (11 ms; *t*(27) = 1.99, *p* = .057). There now also was a trend of unconscious adaptation of interference (10 ms; *t*(27) = 1.74, *p* = .09). For the error analyses, although less prominent, the overall pattern of results for this low *d*′ group also resembled the analyses for the complete group.

In the *conscious condition*, the average proportion of correctly categorized primes (85%) was clearly above chance level (*t*(55) = 17.88, *p*<.001). A measure of prime visibility (*d*′) was again calculated for each subject. Hits or false alarms proportions of zero or one were correct with 0.05. The mean *d′* value was 2.34 which, as can be expected in a conscious condition, significantly differed from 0 (*t*(55) = 18.44; *p*<.001).

## Discussion

In the current study we addressed whether cognitive control can be exerted unconsciously. We used a masked priming paradigm to examine whether conflict adaptation can also be observed when primes remain unconscious. Confirming our predictions, we observed reliable conflict adaptation in both the conscious and the unconscious condition, for both RT and error rate analyses. In contrast to a number of studies suggesting that consciousness is a prerequisite for conflict adaptation (for a review, see [25]), our results are in line with the study of van Gaal et al. [29], who showed that adaptation effects can also be found when the conflicting information remains unconscious. This indicates that, as also suggested by several studies on goal pursuit [18], [19], higher cognitive processes do not always require consciousness. Consequently, our results add to the growing literature showing that many aspects of cognitive control do not seem to have an exclusive link with consciousness (for a review, see [35]).

### Comparing conscious and unconscious conflict adaptation

Next to replicating the findings of van Gaal et al. [29], our second aim was to compare the magnitude of both conscious and unconscious conflict adaptation. Although previous studies observed reduced [29] or non-existent [21] conflict adaptation following unconscious conflict, this might have resulted from using different SOAs in both conditions and/or presenting them intermixed. In our study, we matched for differences in SOA, presented both visibility conditions in separate blocks, and we observed robust conflict adaptation both when primes were presented either clearly visible or heavily masked. The size of the conflict adaptation effect was numerically larger in the conscious compared to the unconscious condition, both in reaction times (26 ms versus 12 ms) and in error rates (5.8% versus 2.6%). However, this difference between conscious and unconscious conflict adaptation was only marginally significant in both cases.

Because congruency effects were much larger in the conscious condition (98 ms) compared to the unconscious condition (14 ms), conscious primes were much more conflicting, and thus we would expect the conscious conflict adaptation effect to be much larger than the unconscious conflict adaptation effect as well. Because this was not the case, a reasonable alternative explanation for the current findings is that not conflict adaptation but another mechanism underlies the trial-by-trial modulation of the congruency effect. Mayr, Awh and Laurey [36] suggested that this effect is a consequence of feature repetitions, rather than monitoring of conflict. This account can explain why we observed similar conflict adaptation in both the conscious and the unconscious condition. However, in an experiment with only two responses, feature repetitions can never completely be ruled out. One way to examine whether feature repetitions might play some role in our study is to test whether the effect survives the exclusion of all trials with stimulus-response repetitions (40.9% of all trials). The results of this analysis closely mirrored our main analyses. Even with the exclusion of all stimulus-response repetitions, the crucial interaction between congruency on the current trial and congruency on the previous trial remained highly significant (*F*(4,52 = 4.80, *p* = .002). As before, there was no interaction with condition (*p* = .40). The conflict adaptation effect was numerically similar to our original analysis, in both the conscious condition (26 ms, *t*(55) = 3.74, *p*<.001) and the unconscious condition (11 ms, *t*(55) = 2.70, *p* = .009). This additional analysis suggests that an interpretation in terms of feature repetitions is not sufficient to explain the current results. However, in order to fully rule out the possibility that future repetitions are the underlying cause of the effect, future research might deploy experiments in which more than two response options are used (e.g., [37]), and test whether the effect survives the exclusion of both prime and target repetitions. In addition, future experiments might map two different stimuli to each response, so that the role of sensory identity priming in conflict adaptation can be examined.

### The origin of adaptation

Our third aim was to examine *the origin* of the adaptation effects. Recently, it was argued that the effect is not caused by conflict (i.e., decreased congruency effects following incongruent trials) but rather the result of increased congruency effects following congruent trials [34]. Schlaghecken and Martini [38] came up with a more general interpretation and proposed that the context on the previous trial (defined by either congruent or incongruent trials) influenced the systems' responsiveness on the current trial, thus challenging the classical interpretation of the sequential modulation of the congruency effect in terms of conflict adaptation [9]. Because we included neutrals trials, we could examine whether our adaptation effects were caused by conflict on the previous trial, by congruent previous trials or whether both trial types added to the effect [38]. Interestingly, our results did not confirm recent observations that adaptation processes are mainly triggered by congruent trials [34], but, on the contrary, suggested that conflict is the main source of conscious and unconscious adaptation effects [9]. A possible way to explain this apparent discrepancy is to assume that the particular type of neutral trials that are used can seriously alter conclusions [39]. Whereas in previous studies it could not be assured that neutral trials were actually neutral [34], the neutral trials in our study were responded to slower than congruent trials, but faster than incongruent trials, which indicates that they were really neutral. In sum, our results support an interpretation of the sequential modulation of the congruency effect in terms of conflict adaptation.

### Disentangling conflict adaptation: Interference and Facilitation

Next to this examination of the origin of adaptation, we also examined which trials are most affected by the conflict-evoked adaptation. We examined whether the observed conflict adaptation effects were caused by adaptation of facilitation (i.e., faster responses and/or less errors on congruent than on neutral trials) following conflict or adaptation of interference (i.e., slower responses and/or more errors on incongruent trials than on neutral trials) following conflict. Next to the reliable conscious and unconscious conflict adaptation, we found a clear pattern of adaptation of interference. Both in the conscious and unconscious condition interference effects were found to be reduced following incongruent trials. Following conflict, there is an attention shift towards the relevant information (i.e., the target; [40]) or away from the irrelevant information (i.e., the prime; [41]), which is primarily beneficial on current incongruent trials, which are most hampered by the conflicting information.

Interestingly, whereas the error rates in the conscious condition confirmed this pattern of adaptation of interference, the results of the error rates in the unconscious condition showed adaptation of facilitation. The performance on congruent trials improved after unconscious conflict, while the performance on incongruent trials remains stable. This unexpected finding suggests that there might be a different form of adaptation to unconscious conflict for errors and for reaction times. This suggests that although conscious and unconscious conflict adaptation share the same origin (i.e., conflict), this conflict may trigger different adaptation processes in the error rates dependent on conscious awareness. This finding is in line with our recent observation that conscious priming is mainly caused by interference and unconscious priming by facilitation [42]. However, because our experiment was not difficult, average error rates were rather low (conscious condition: 4.2%; unconscious condition: 2.7%). Therefore, we must be cautious in interpreting these effects, because they are based on a very small amount of observations. Moreover, because the possibility of unconscious conflict adaptation is heavily debated, and the proof in favor of it is scarce [29], the suggestion that conscious and unconscious adaptation might be exerted differentially, must be treated with serious precaution, and needs to be confirmed in future research.

### Alternative interpretations

Although our results support the classical interpretation of the Gratton effect in terms of conflict adaptation [9], several alternative interpretations of our results need to be taken into consideration. For example, according to Kinoshita and colleagues [43], the size of congruency effects is mainly determined by recent trial difficulty. Since incongruent trials are more difficult than congruent trials, this account predicts reduced congruency effects following incongruent (i.e., difficult) trials. If subjects are consciously aware of the difficulty of a trial, although being unaware of the prime itself, based on this account our results can be explained in terms of adaptation to a consciously experienced by-product of the unconscious conflict. Since congruency and difficulty are confounded in the current study, it is not possible to disentangle these two possible sources of adaptation. Apart from an interpretation in terms of adaptation to recent trial difficulty, a more low-level explanation would explain our results in terms of passive decay of facilitation. Since the neural traces created by unconscious primes are very weak and short-lived [30], there tends to be a fast passive decay of these traces, which results in reduced congruency effects in bins with the slowest reaction times [39]. Because we tend to respond fast following congruent trials [44] and slow following incongruent trials [20], the smaller congruency effects following incongruent trials might have been caused by passive decay of primes on these slow trials. To exclude this possibility, we examined whether the unconscious conflict adaptation effect was only apparent in the bins with slow reaction times. For this analysis, we selected all congruent and incongruent trials from the unconscious condition, which were preceded by congruent or incongruent trials. Subsequently, we rank ordered the data according to reaction time, and then divided the data into percentiles, by aggregating ten bins over subjects. Subsequently, we ran an ANOVA with the factors congruency on the current trial and congruency on the previous trial as within-subjects factors, and percentile as a covariate. As expected, this analysis showed an interaction between congruency on the current trial and congruency on the previous trial (*F*(1,8) = 9.45, *p* = .015). Crucially, the interaction was not modulated by the percentile (*F*<1). When looking at the data, the conflict adaptation effect proved to be stable over different bins (from fastest to slowest percentile: 4 ms, 10 ms, 17 ms, 19 ms, 19.5 ms, 18 ms, 13 ms, 13 ms, 7.3 ms, 18 ms).

### Conclusion

In short, although some alternative explanations for our current results are worth pursuing in future experiments, an interpretation of the data in terms of adaptation to conflict is currently preferable as discussed above. We conclude that conflict adaptation is possible when the conflicting information remains unconscious, confirming the findings of van Gaal et al. [29]. Thus, conflict adaptation, as a prevailing expression of cognitive control, does not seem to be a function exclusively reserved for consciousness. This observation contributes to the search for the limits and possibilities of unconscious processing and can be helpful to further unravel the mystery of the function of consciousness.
